# Graphene‐Based Technologies for Tackling COVID‐19 and Future Pandemics

**DOI:** 10.1002/adfm.202107407

**Published:** 2021-09-16

**Authors:** Shaila Afroj, Liam Britnell, Tahmid Hasan, Daria V. Andreeva, Kostya S. Novoselov, Nazmul Karim

**Affiliations:** ^1^ Centre for Print Research The University of West of England Bristol BS16 1QY UK; ^2^ Graphene Engineering and Innovation Centre (GEIC) The University of Manchester Manchester M13 9PL UK; ^3^ Department of Environmental Science and Engineering Bangladesh University of Textiles Tejgaon Dhaka 1208 Bangladesh; ^4^ Department of Materials Science and Engineering National University of Singapore Singapore Singapore; ^5^ Institute for Functional Intelligent Materials National University of Singapore Singapore Singapore; ^6^ Chongqing 2D Materials Institute Liangjiang New Area Chongqing 400714 China

**Keywords:** 2D materials, biosensors, COVID‐19, filtration, graphene, personal protective equipment, wearables

## Abstract

The COVID‐19 pandemic highlighted the need for rapid tools and technologies to combat highly infectious viruses. The excellent electrical, mechanical and other functional properties of graphene and graphene‐like 2D materials (2DM) can be utilized to develop novel and innovative devices to tackle COVID‐19 and future pandemics. Here, the authors outline how graphene and other 2DM‐based technologies can be used for the detection, protection, and continuous monitoring of infectious diseases including COVID‐19. The authors highlight the potential of 2DM‐based biosensors in rapid testing and tracing of viruses to enable isolation of infected patients, and stop the spread of viruses. The possibilities of graphene‐based wearable devices are discussed for continuous monitoring of COVID‐19 symptoms. The authors also provide an overview of the personal protective equipment, and potential filtration mechanisms to separate, destroy or degrade highly infectious viruses, and the potential of graphene and other 2DM to increase their efficiency, and enhance functional and mechanical properties. Graphene and other 2DM could not only play a vital role for tackling the ongoing COVID‐19 pandemic but also provide technology platforms and tools for the protection, detection and monitoring of future viral diseases.

## Graphical abstract

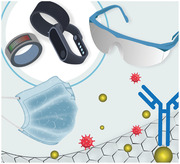

## 1 Introduction

The emergence of coronavirus disease 2019 (COVID‐19) has been the greatest threat of this century to the worldwide community, due to the global spread and severity of the disease. As of Aug 2021, globally there have been ≈214.5 million confirmed cases of COVID‐19 in 170 countries, and ≈4.5 million deaths reported to World Health Organization (WHO).^[^ ^1^ ^]^ Such an extraordinary situation is not only affecting the health of the population but also has clear significant impact on global economy, political and social aspects. Additionally, the dramatic impacts on financial markets all over the world have caused unemployment, business failure, hardship for certain industries including tourism and aviation, and created an unprecedented level of risk for the investors. Many scientists have already shifted focus of their research into developing novel technologies to combat COVID‐19, and several vaccines have already been developed. According to data from International clinical Registry Platform, 323 randomised controlled trials and 134 non‐randomised studies of the COVID‐19 vaccines are currently ongoing,^[^ ^2^ ^]^ and a total of 4.9 billion vaccine doses have been administered (August 2021).^[^ ^1^ ^]^ However, time will be needed to vaccinate the mass population worldwide. Additionally, vaccines may provide short‐term immunity and the introduction of new COVID‐19 variants (e.g., alpha, beta, gamma, and delta) has made the situation worse, as they spread quicker than previous variants, and are associated with a higher risk of death.^[^ ^3^ ^]^ While scientists turned their attention into developing new technologies to combat COVID‐19,^[^ ^4^, ^5^ ^]^ as evident from hundreds‐of‐thousands COVID‐19 related research journal papers published so far since the outbreak, there remains a lack of robust technologies that can facilitate detection, monitoring and protection from COVID‐19.

**Figure 1:**
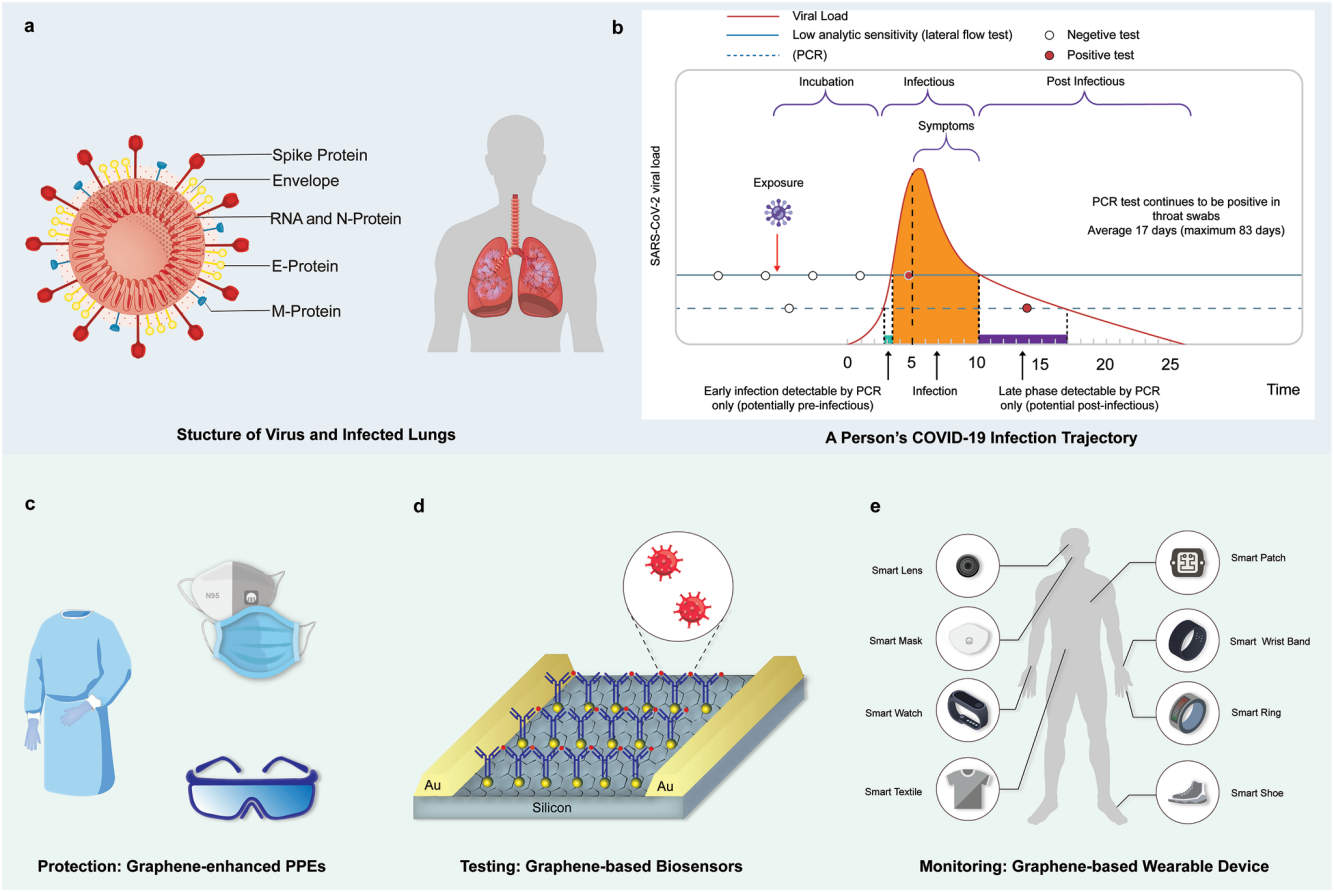
COVID‐19 and potential graphene‐based technologies for tackling COVID‐19 virus. a) The structure of a COVID‐19 virus and transmission (via water, air, food, blood, and touch) to infect lungs of a human body. b) A COVID‐19 patient's infection trajectory (blue lines) in context of two surveillance regimens (circles), where the lateral flow test is more likely to detect infection during the transmission window (shading) than PCR, despite its lower analytic sensitivity. Reproduced with permission.^[^ ^6^ ^]^ Copyright 2020, Massachusetts Medical Society. c) Potential graphene‐based PPEs for the protection against viruses. d) Prospective graphene‐based biosensors to enable rapid detection of COVID‐19 and e) Future graphene‐based wearable technologies for continuous monitoring of physiological conditions.

Graphene, since its isolation in 2004, has received much attention from the researcher community, owing to its incredible mechanical, thermal, electrical and other properties.^[^ ^7^ ^]^ In addition, the isolation of graphene has unveiled a wide range of graphene‐like 2D materials (2DM) with outstanding properties. Such materials could be assembled into one multilayer stack and create heterostructure‐based multifunctional devices, enabling extraordinary control of their properties and functionality. Graphene has already demonstrated potential for biosensing,^[^ ^8^, ^9^, ^10^ ^]^ wearable electronics,^[^ ^11^, ^12^, ^13^ ^]^ and filtration^[^ ^14^, ^15^ ^]^ which could be used for the detection, continuous monitoring and the protection from the infectious diseases including COVID‐19 (**Figure** **1**a–e).^[^ ^16^, ^17^, ^18^ ^]^ The incredible properties of graphene could be exploited to manufacture next generation personal protective equipment (PPE) or wearable e‐textiles for fighting COVID‐19. For instance, robust nano‐scaled multifunctional devices can be designed using a combination of electrically and optically active 2D materials and stimuli‐responsive biomaterials to perform bio‐detection, sensing, self‐cleaning, and self‐regeneration functions.

Here, we explore how graphene, other 2DM and composites could potentially offer alternative or complementary solutions for detecting and monitoring COVID‐19. We also discuss their role for developing novel biosensors and wearable technologies, which can be designed to detect virus antigens or antibodies, as well as continuous monitoring of COVID‐19 symptoms, thus helping to contain the pandemic by controlling the reproduction rate (*R*). We also review the current PPE and virus filtration techniques and discuss the potential of 2D materials for manufacturing sustainable and highly effective anti‐viral PPE and virus flirtation techniques. Finally, we investigate future research directions for graphene‐based technologies to develop novel detection and protection technologies to combat COVID‐19 outbreaks as well as any future virus outbreaks.

## 2 The Detection and Continuous Monitoring: Test and Trace

The detection of COVID‐19 is considered as a critical step for the isolation and treatment of COVID‐19 infected patients. In addition, the rapid and extensive testing helps contact tracing of close contacts of anyone who has tested positive for COVID‐19, and isolate them to stop the spread of the virus. Thus, the test and trace service enable control of the reproduction rate (*R*), and helps governments set strategies and take necessary control measures including local or national lockdowns to contain the pandemic and save lives. Currently, several COVID‐19 test methods are available on the market,^[^ ^19^ ^]^ which can broadly be categorized into viral tests (to detect the current infection) and antibody testing (also known as a serologic test). Two types of viral tests can be used: nucleic acid amplification tests (NAATs) and antigen tests. The WHO and US Centres for Disease Control and Prevention recommend real‐time reverse transcription polymerase chain reaction (PCR) for diagnosis of COVID‐19 infection which involves the detection of viral RNA using NAATs.^[^ ^20^ ^]^

PCR is the most widely used gold standard for testing COVID‐19 infections due to its high sensitivity and specificity.^[^ ^21^ ^]^ However, a PCR test is a time‐consuming process which requires a sophisticated laboratory and machines, trained personnel and logistics for transporting samples. In addition, the possibility of obtaining false‐negative results from PCR tests can be as high as ≈29%,^[^ ^22^ ^]^ which is a growing concern. Furthermore, delays in obtaining PCR results leave a window in which the shedding of infectious virus occurs and the control of onward transmission remains a challenge. Alternative antigen lateral flow tests provide rapid results but suffer from inferior sensitivity and specificity to PCR. Nevertheless, rapid antigen tests can provide useful information as point‐of‐care testing, thereby interests in their performance are growing with a particular focus on the sensitivity and overall specificity.^[^ ^23^ ^]^ The antibody‐based test methods are promising but it can take weeks or months for antibodies to appear after the initial exposure. Therefore, there remains an unmet need for a rapid and low‐cost testing method with excellent sensitivity and specificity that would enable widespread population‐level testing at scale, as well as monitoring of the COVID‐19 infection. Graphene and other 2DM‐based biosensors and wearable technologies could potentially offer alternative or complementary alternative solutions for detecting and continuous monitoring of COVID‐19 and other viruses.

### 2.1 Biosensors

Biosensors have seen significant interest in recent years due to their ability to provide rapid, sensitive, portable, miniaturized and inexpensive alternative platforms to traditional diagnostics systems.^[^ ^24^ ^]^ A typical biosensor consists of three parts: “bioreceptors” unit (for example, enzyme, antibody or DNA) in combination with ion conductive materials that recognize the analyte, a physio‐chemical signal transducer (for example, electrochemical, optical or piezoelectric) that translates this bio‐recognition event into a useful signal, and a reader device (Figure 1d).^[^ ^25^ ^]^ Such devices could potentially detect viruses including COVID‐19 with remarkable sensitivity and specificity, which is extremely important to determine the usefulness and reliability of test results.

Proteins or DNA are usually attached to robust functional synthetic materials to form a bio‐detection unit. Such composite surfaces provide high sensitivity, fast response, and reversibility of bio‐recognition events.^[^ ^26^ ^]^ Bio‐recognition as other biological processes operates with ionic current.^[^ ^27^ ^]^ Therefore, ion conductive, and ion and electron conductive polymers are commonly used for the formation of bio‐sensitive surfaces.^[^ ^28^ ^]^ The enhanced sensing functionality of synthetic and natural polyelectrolytes comes from the fact that the charge state of the polyelectrolyte depends strongly on external conditions. Upon changing such conditions, the macromolecules are able to change their charged state releasing ions and conformation, creating a signal that can be used for transduction.^[^ ^29^ ^]^ Recent advances in transduction systems, nanotechnology and genetic engineering have significantly improved the detection performance of biosensors.^[^ ^30^, ^31^ ^]^ Depending on the transduction systems, biosensors can be categorized into three main types: electrochemical, optical, and piezoelectric biosensors. Electrochemical biosensors convert the chemical signal into an electrical signal and monitor changes in charge distribution over the transducer surface based on potentiometric, amperometric, or impedimetric transduction principles. An optical signal is emitted in optical biosensors, which is directly proportional to the concentration of the analyte. Piezoelectric biosensors measure the alterations in resonate frequency due to a mass bound on the piezoelectric crystal surface.

Biosensors for detecting COVID‐19 and other viruses can be designed to detect virus antigens, antibodies produced against a virus or the viral genome.^[^ ^33^ ^]^ Indeed, it is easier to detect viral antigens as they are displayed on the outer surface of the virus and can strongly be attached to receptors or antibodies carried by the biosensor. The majority of biosensors developed so far, however, have focused on the detection of the influenza viruses.^[^ ^34^, ^35^, ^36^ ^]^ This is mainly due to the fact that the influenza virus has many harmful subtypes with annual morbidity and mortality worldwide. These circumstances have motivated the development of technologies for fast diagnosis with the reliability found in biosensors. Nevertheless, biosensors have been developed to detect SARS‐CoV as a faster and more sensitive alternative to the RT‐qPCR and serological tests.^[^ ^37^, ^38^, ^39^ ^]^ The recent emergence of deadly and highly infectious SARS‐CoV‐2 (COVID‐19) viruses has forced scientists to develop alternative diagnostics for quick and sensitive detection of COVID‐19 followed by contact tracing and containment strategies. Furthermore, biosensing devices developed for the detection of Influenza and SARS‐CoV viruses could be used as models for developing similar devices detecting COVID‐19, as the biological materials (e.g., antibodies, antigens, DNA, etc.) are essentially the same.^[^ ^40^ ^]^ Recent studies^[^ ^20^, ^41^, ^42^, ^43^, ^44^, ^45^, ^46^ ^]^ have reported several promising nanotechnology‐based biosensing devices for diagnosing COVID‐19 via both nucleic acid‐ and antigen‐based detection, including platforms based on gold nanoparticles, carbon nanotubes and quantum dots. However, the use of such nanomaterials is problematic due to their inconsistent signal amplification and the presence of metallic impurities.^[^ ^47^ ^]^

Graphene and graphene‐like 2D materials including transition metal dichalcogenides, graphitic carbon nitride, MXenes, hexagonal boron nitride (h‐BN), and transition metal oxides, have shown great promise as transduction system and supporting substrates for fabricating next generation biosensors.^[^ ^48^, ^49^ ^]^ Extremely high surface area, coupled with broad range of electronics and optical properties, make graphene and graphene‐like 2D materials ideal for biosensing applications. Additionally, 2D materials can be combined easily in one multilayer stack to fabricate heterostructure devices with tuneable functionalities and properties. Furthermore, graphene and graphene‐like 2D materials can be tailored to selectively respond to specific analytes with extremely high sensitivity via modifying their surface functionalities or defect engineering, which is particularly attractive for rapid and easy‐to‐use virus detection. For example, oxygen‐containing functional groups of graphene oxide (GO) and reduced graphene oxide (rGO) can be fine‐tuned, which is often crucial for binding interactions between graphene derivatives and biomolecules.^[^ ^50^ ^]^ Indeed, graphene‐based innovative biosensing devices have already been reported and summarised in several studies.^[^ ^51^, ^52^, ^53^, ^54^, ^55^ ^]^ Current 2D materials‐based biosensors can be categorized as: electrode‐based devices, including electrochemical, and field‐effect transducers, and optical systems mainly relying on sensing by fluorescence, surface plasmon resonance (SPR), and surface‐enhanced Raman scattering.^[^ ^56^ ^]^ Among them, single to few layers graphene‐based field‐effect transistors (FET) have shown promise due to their ultra‐sensitivity and low‐noise detection, thereby providing quick measurements even in the presence of small amounts of analyte.^[^ ^57^ ^]^ Graphene‐based electrical sensors provide several advantages including small size, large surface area, fast electron transfer, fast response time, high sensitivity, and reduced surface contamination. However, they are limited to measure current changes only, low spatial resolution and potentially damaging samples, which may affect results.^[^ ^58^ ^]^ Similarly, graphene‐based optical sensors provide high spatial resolution, wide and deep detection range, high sensitivity and precision, accurate and fast detection. However such sensors can suffer from low light absorption rate, aggregation, and precipitation of high concentrated sample, which may affect the optical detection.^[^ ^59^ ^]^

**Figure 2:**
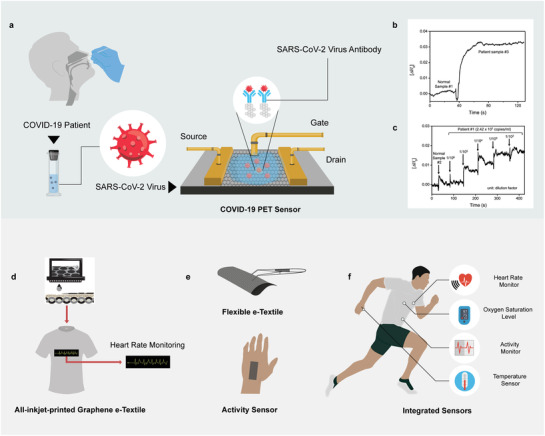
Graphene‐based biosensors and wearable e‐textiles for tackling COVID‐19. a) Graphene‐based FET sensor for detecting SARS‐CoV‐2 spike protein from COVID‐19 patients. b,c) Comparison of response signal between control and patient samples. Reproduced with permission.^[^ ^32^ ^]^ American Chemical Society. d) Fully inkjet‐printed graphene‐based wearable e‐textiles for monitoring heart rate. e) Flexible and wearable graphene‐based textiles sensors for monitoring human activity. f) Concept future wearable e‐textiles garment for remote monitoring of signs for early detection of asymptomatic and pre‐symptomatic cases of COVID‐19 and other viruses.

Since the COVID‐19 outbreak, researchers have turned their attention into developing graphene‐based biosensors (**Table** **1**). Recently, a graphene‐based FET biosensor was developed for detecting COVID‐19, using spike protein antibodies as the detection probe (**Figure** **2a**).^[^ ^32^ ^]^ In this work, the sensor performance was evaluated using an antigen protein, cultured virus, and nasopharyngeal swab specimens from both COVID‐19 patients and control subjects, which demonstrated the detection of SARS‐CoV‐2 spike protein at concentrations of 1 fg mL^−1^ in phosphate‐buffered saline and 100 fg mL^−1^ clinical transport medium. Additionally, the graphene‐based FET sensor clearly discriminated between patient and control samples, and responded to patient samples diluted as much as 1:1 × 10^5^ (242 copies mL^−1^; Figure 2b,c). Although the limit of detection (LOD) of the developed COVID‐19 FET sensor is slightly higher than the current molecular diagnostic test of COVID‐19 (50–100 copies mL^−1^),^[^ ^64^ ^]^ the LOD is low enough for practical use. The reported system proved to be advantageous for detecting the COVID‐19 virus from clinical samples without any pre‐processing and with a large dynamic range, but further development of novel materials for FET sensors would be needed for more accurate detection by reducing noise. In another study,^[^ ^61^ ^]^ an MXene–graphene FET sensor was developed for detecting influenza and SARS‐CoV‐2 viruses. In this work, an ultra‐sensitive virus‐sensing transduction material was developed, which combines the high chemical sensitivity of MXene and the continuity of large‐area high‐quality graphene. The developed FET sensors provide low LOD (125 copies mL^−1^ for the influenza virus and 1 fg mL^−1^ for the recombinant 2019‐nCoV spike protein), as well as faster response time (≈50 ms) than existing detection techniques. The reported low‐cost, ultrasensitive, fast‐responding, and high specific 2D materials‐based COVID‐19 sensor will further advance the biosensing field by innovating novel devices for detecting highly infectious viruses including COVID‐19.

**Table 1:** Summary of recently developed graphene‐based biosensors for detecting COVID‐19

Detected element	Sensing material and sensor type	Limit of detection (LOD)	Ref.
SARS‐CoV‐2 spike protein	Graphene (FET sensor)	Culture medium: 1.6 × 10^1^ pfu mL^−1^ Clinical samples: 2.42 × 10^2^ copies mL^−1^	^[^ ^32^ ^]^
SARS‐CoV‐2 spike protein	WSe_2_ (FET sesnor)	25 fg µL^−1^ in 0.01X phosphate‐buffered saline (PBS).	^[^ ^60^ ^]^
Influenza virus and 2019‐nCoV	MXene‐graphene (FET Sensor)	125 copies mL^−1^ for the influenza virus 1 fg mL^−1^ for the recombinant 2019‐nCoV spike protein	^[^ ^61^ ^]^
SARS‐CoV‐2	Graphene (nanoresonator sensors)	10 copies per test	^[^ ^62^ ^]^
SARS‐CoV‐2	BK_7_/Au/PtSe_2_/Graphene (surface plasmon resonance (SPR) biosensor)	Sensitivity: 183.33°/refractive index unit (RIU) in SPR angle (θ_SPR_)	^[^ ^63^ ^]^

Thanks to excellent electrical properties and large surface area, graphene and other 2DM will be better candidate for fabricating next generation FET‐based biosensors to detect infectious viruses including COVID‐19. Graphene provides higher electron mobility but suffers from current leakage and reduced sensitivity due to its zero‐band gap. In contrast, a larger band gap can be achieved with other 2DM such as MoS_2_, WSe_2_, and MnO_2_, which can be switched between semiconducting and insulating states. Thus, current leakage can be minimised, and higher sensitivity can be achieved. Furthermore, the surface modification or functionalisation of 2DM can be carried out to tailor the band gap and carrier concentration. Nevertheless, the successful commercialisation of such biosensors will also depend on the life‐time, storage, and operation time, which need to be considered at the design stage of a biosensors.^[^ ^56^ ^]^

For biosensing, it is important to propose new composite materials that can operate with both ionic and electronic currents. Such a 2D composite can be built using polyelectrolytes, GO and electronically conductive 2D materials. GO is a derivative of graphene that is widely used for the design of biorecognition units of biosensors.^[^ ^65^ ^]^ GO has an ideal compatibility with soft bio‐interfaces and demonstrates similar ionic transport properties as polyelectrolytes.^[^ ^66^ ^]^ So it can be used as an ion conductive component in composites and also it can be decorated by polyelectrolytes and biomolecules to achieve additional functionality.^[^ ^67^ ^]^ It was shown that the change of ionic concentration in the interior of the GO‐polyelectrolyte composite material can be used as gating for the building of transistors operating with ionic current.^[^ ^68^ ^]^ This functionality can be used for sensing of the presence of the virus.^[^ ^69^ ^]^ The properties of such composite transistors depend on electrochemical environment. The positively charged surfaces made of cationic polyelectrolytes will attract biological objects, including COVID‐19. Due to electrostatic interactions between viruses and polyelectrolytes, we can achieve a switch in ionic current in the transistors (detection and signalling function).^[^ ^69^ ^]^

### 2.2 Wearable Devices

According to recent NHS (UK) data (2019), approximately 307 million patient consultations take place every year at GP surgeries. Additionally, outpatient visits at hospitals have approximately doubled over the past decade (from 54 to 94 million per year), adding extra expenditure of ≈£8 billion per year to NHS costs.^[^ ^70^ ^]^ To tackle this ever‐increasing pressure on healthcare system, and provide quality healthcare services to the mass population, the NHS has published a long‐term plan with a vision to move towards digital health services.^[^ ^70^ ^]^ Wearable technologies such as activity trackers and smartwatches will play significant role in implementing such a plan via early prediction, and continuous monitoring and tracking of diseases. Such wearable devices can provide greater understanding into our health and well‐being,^[^ ^71^ ^]^ by collecting real‐time physiological data instead of an occasional snapshot during medical appointments. Furthermore, it allows detection of deviations in health data from an individual's “typical” baselines, which is fundamentally a different approach from the current practice that is based on population statistics.

Now as the current COVID‐19 pandemic is ongoing, the potential of such wearable devices has become increasingly apparent. The main symptoms of coronavirus (COVID‐19) include a high temperature (98%), a new continuous cough (65%) and shortness of breath (55%).^[^ ^72^ ^]^ Additional symptoms include silent hypoxemia (an abnormally low level of oxygen in the blood), and a loss or change of sense of smell or taste. Therefore, consumer‐grade wearable devices which are readily available and interoperable with smartphones, could potentially be used for continuous monitoring of vital signs, although they have limitations in accuracy and measurement modalities.^[^ ^73^ ^]^ Three most well‐known commercial wearable devices are: FitBit, Apple Watch, and Oura Ring, which offer heart rate and physical activity measurements but not body temperature, respiratory rate or oxygen saturation level. Additionally, FitBit and Oura Ring have not received U.S. Food and Drug Administration approval yet for remote monitoring of patients’ physiological conditions.^[^ ^73^ ^]^ Projects like Snap40 were kicked off well before COVID‐19 outbreak (in 2017 but report yet to be published) involving 250 NHS patients, where a single wearable device was attached on the upper arm to continuously monitor skin temperature, respiratory rate, oxygen saturation, relative change in systolic blood pressure, heart rate, and body movement.^[^ ^74^ ^]^

Since the COVID‐19 outbreak, researchers have already started investigating the use of wearable technology for early detection of asymptomatic and pre‐symptomatic cases of COVID‐19. In a recent study,^[^ ^75^ ^]^ pre‐symptomatic cases of COVID‐19 were detected from the smartwatch data including heart rate, sleep time and number of daily steps via investigating correlations between different metrics, and detecting unusual physiology. The health data of 32 COVID‐19 infected individuals out of 5,300 participants was collected from smart devices including Apple Watch and FitBits. The elevated resting heart rates (relative to individual's baseline), and the increased heart rate in relation to number of steps were observed. It was found that 26 out of 32 (81%) COVID‐19 infected participants displayed aberrant physiological signals 4–7 days prior to onset of symptoms or diagnosis. However, such study fails to differentiate COVID‐19 from other viral infections, and also questions its diagnostic performance due to a lack of additional physiological information (depends only on heart rate), effects of environmental, behavioural and other external factors (such as drug, alcohol, travel, stress, diet, and other health conditions).

In another study,^[^ ^76^ ^]^ it was investigated that how smartwatch data and self‐reported symptoms can help to detect COVID‐19. The data collected from a pool of over 30 000 participants shows that the resting heart rate data alone is not a major differentiator between COVID‐19 positive and negative cases in the area under the curve (AUC) of the receiver operator characteristic (ROC curve was 0.52), whereas accuracy improves as sleep data and activity metrics were added to resting heart rate data (AUC increased to 0.72), and improves further (AUC increased to 0.80) as self‐reported symptoms were addressed. However, this methodology is also unable to differentiate COVID‐19 from other viral infections, and does not take other physiological, behavioural, environmental and external factors into consideration. Additionally, such devices are bulky and rigid, and most importantly not comfortable to wear.

With the aim to achieve wearer's comfortability, researchers have put efforts into developing on‐body skin‐integrated sensors (soft electronic tags/patch that couple intimately to the skin) that can measure respiratory biomarkers (such as cough intensity/frequency/sound, respiratory effort and rate).^[^ ^77^ ^]^ A company called VitalConnect (San Jose, CA) has introduced a single‐use chest patch in nursing homes to track and continuous monitoring of heart rate and respiratory rate of vulnerable elderly patients.^[^ ^73^ ^]^ In another project by Imperial College (London, UK), a temporary NHS facility was setup near Heathrow airport to monitor heath data of quarantined travellers. Wearable sensors were used for collecting heath data such as temperature, heart rate, and respiratory rate of individuals in quarantine.^[^ ^78^ ^]^

Wearable e‐textiles could provide alternative to existing bulky and rigid devices, as they are less visible, and offer more flexibility and comfort to the wearer. Currently, most of the wearable e‐textiles in the market are based on metal inks such as Ag, Cu and Au, due to their high electrical conductivity (usually around 10^5^ S m^−1^) compared to polymer‐based inks such as PEDOT, polypyrroles and polyanilines (usually around 10^2^ S m^−1^). However, metal‐based inks are non‐biocompatible, expensive, harmful to the environment and often need higher sintering temperature, which may damage heat‐sensitive textiles.^[^ ^79^ ^]^ Therefore, there remains a need for developing complementary wearable technologies in order to predict, detect and monitor COVID‐19 infections.

Recently, graphene‐based smart wearable e‐textiles^[^ ^80^ ^]^ have received great attention from the researcher community due to graphene's exceptional properties such as outstanding electrical and thermal properties. Among them, rGO‐based wearable e‐textiles are particularly of interest,^[^ ^13^, ^79^, ^80^, ^81^, ^82^ ^]^ due to their better adhesion to textiles via possible covalent and hydrogen bonding between functional groups of textiles and residual oxygen‐containing functional groups of rGO. Thus, rGO‐based textiles show better washing stability, which is one of the key requirements for wearable e‐textiles. However, rGO suffers from poor electrical conductivity due to the defects in the crystal structure and partial restoration of the sp2 structure of graphene during the reduction process, and may not be suitable for some applications where very high electrical conductivity is required. Highly conductive and machine‐washable graphene‐based e‐textiles can be produced via scalable pad−dry−cure method with subsequent roller compression and a fine encapsulation of graphene flakes.^[^ ^83^ ^]^

In our previous works,^[^ ^13^, ^81^, ^83^, ^84^ ^]^ it was demonstrated that how various sensors (temperature, activity monitoring and heart rate) based on graphene textiles can contribute to individual's health and well‐being. In the reported works, we particularly focused and put our efforts on developing highly scalable, washable and flexible wearable e‐textiles platforms with improved electrical conductivity and strength, better sensitivity, breathable and comfortable to wear, as well as environmentally friendly. Such platform technologies can be used for developing wearable garments with multimodal and/or multiplexed measurements capability, which may ultimately improve the performance and reliability of individual sensors by accommodating additional information.^[^ ^85^ ^]^ Thus graphene‐based wearable e‐textiles could potentially be used for continuous on body monitoring of COVID‐19 symptoms.

In our other work^[^ ^86^, ^87^ ^]^ all‐inkjet‐printed graphene‐based wearable e‐textiles was reported that can be used as a non‐invasive heart monitoring device. The methodology includes inkjet printing of graphene‐based conductive inks on a rough and porous textiles surface for the first time. Inkjet printing enables localised deposition of an exact amount of graphene‐based conductive materials at a predefined location. The use of water‐based, non‐toxic and bio‐compatible graphene inks could potentially open up the prospect of manufacturing next generation smart e‐textiles for personalised healthcare applications including continuous monitoring of COVID‐19 infection. In our other works,^[^ ^13^, ^81^, ^83^, ^84^ ^]^ it was demonstrated that how various sensors (temperature, activity monitoring and heart rate) based on graphene textiles can contribute to individual's health and well‐being. In the reported works, we particularly focused and put our efforts on developing highly scalable, washable and flexible wearable e‐textiles platforms with improved electrical conductivity and strength, better sensitivity, breathable, and comfortable to wear, as well as environmentally friendly. Such platform technologies can be used for developing wearable garments with multimodal and/or multiplexed measurements capability, which may ultimately improve the performance and reliability of individual sensors by accommodating additional information.^[^ ^85^ ^]^ Thus graphene‐based wearable e‐textiles could potentially be used for continuous on body monitoring of COVID‐19 symptoms.

## 3 Protection from Viruses

### 3.1 Personal Protective Equipment (PPE)

Before the COVID‐19 pandemic, the world has seen four more pandemics since 1900: the “Spanish Flu” in 1918 (caused by A(H1N1) virus), the “Asian Flu” in 1957 (caused by A(H2N2) virus), the “Hong Kong Flu” in 1968 (caused by A(H3N2) virus) and “Swine Flu” in 2009. Such highly infectious viruses have taken millions of lives, as they are highly transmittable while the infected person may not show any symptoms at all. The lungs cells are affected first, once infected with COVID‐19 viruses, which could kill lung cells as well as cause malfunctioning of these cells (known as “syncytia”). Recent data from COVID‐19 patients shows that clotting chemicals in the blood can increase up to 400% higher than normal levels. Additionally, the inflammation in some patients may eventually weaken the immune system, causing damage to the rest of the body, especially in blood vessels, liver, kidneys, and lungs.^[^ ^91^ ^]^ Such pathogens mainly transmit between humans by three primary routes: direct contact, airborne transmission and respiratory droplet transmission. PPE can prevent or reduce such contacts and droplet exposures by creating a barrier between human body and the pathogens.^[^ ^92^ ^]^

PPE is considered to be a critical component due to its ability to protect users from droplets from coughs, sneezes, and aerosol‐generating procedures, in addition to other contaminated body fluids and surfaces from infected patients.^[^ ^88^ ^]^ In general, protective aprons, gowns or coveralls, masks or respirators, gloves, goggles or face shields are used as PPE for healthcare workers. There are three basic factors that need to be addressed to select effective PPE. First, the type and amount of bodily fluid the wearer might be exposed to, and how these fluids might transmit to the body. Second, durability and appropriateness of the PPE for the particular task. And third, the fit of the PPE to the wearer. Gloves are the widely used PPE to protect hands, which are usually made of natural or synthetic rubber of sterile and nonsterile types. It is important that the gloves used for protective purposes are tear/damage‐free, fit well and comfortably. Protective aprons, gowns or coveralls are commonly used to protect skin and other clothing. They should fully cover the torso and sleeve up to the wrist comfortably and properly. To protect all or parts of the face (nose, mouth, and eyes) a combination of PPE types such as masks or respirators, goggles, and face shields are used. Protective goggles should fit softly over and around the eyes to protect eyes. Masks should fully cover the nose and mouth to prevent airborne transmission and respiratory droplet transmission. Respirators such as N95, N99, or N100 which are capable of excluding particles that are less than 5 µm in diameter are widely used as PPE by the healthcare workers. When high‐risk aerosol‐generating procedures (such as bronchoscopies) are being performed, a higher level of respiratory protection is used where powered air‐purifying respirator (PAPR) purifies the air. In some cases, face shields are used instead of googles and mask to cover the full face (from forehead to the chin and wrap around the sides of the face).

Typically, protective medical wear is made of synthetic fibers because of their better liquid barrier properties. In addition, they are fabricated as woven, knitted or of non‐woven structure. Disposable medical textiles such as surgical caps, surgical gowns and surgical masks are mainly made of nonwoven fabrics, as such fabrication technique offers scalable and economical production with superior sterility and infection control than other processes. Such disposable medical textiles generally composed of polypropylene fibers, and constructed as spunbond–meltblown–spunbond. On the other hand, medical protective clothing like scrubs is fabricated by weaving cotton or polyester/cotton.

**Figure 3:**
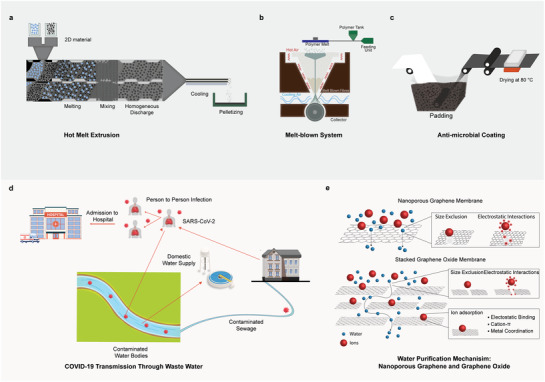
Graphene‐based technologies for sustainable PPE and water purification. a) Hot‐melt extrusion process for melt‐mixing of graphene and other 2DM to fiber polymers. b) Melt‐blown nonwoven fabric manufacturing technique for personal protective clothing. c) The application of antimicrobial finish on textiles via pad–dry–cure technique. Reproduced with permission.^[^ ^88^ ^]^ Copyright 2020, American Chemical Society. d) The potential sources and pathways of COVID‐19 in water systems. Reproduced with permission.^[^ ^89^ ^]^ Copyright 2020, Elsevier B.V. All rights reserved. e) Nano‐porous graphene and GO membrane for water purification. Reproduced with permission.^[^ ^90^ ^]^ Copyright 2015, Royal Society of Chemistry.

Specialist finishes (such as a fluid repellent finish) can be applied to all these mentioned disposable or reusable medical textiles, to further improve their protection capacity from body fluid or hazardous substance contamination. Additionally, antimicrobial finishes can be highly effective against highly transmittable deadly pathogens (such as coronavirus, hepatitis B virus, hepatitis C virus, ebola virus, and human immunodeficiency virus) by preventing infections either by killing or by inhibiting viruses and bacteria.^[^ ^88^ ^]^ Graphene materials (GMs) such as graphene, GO, rGO, and graphene quantum dots have been investigated as a new type of broad spectrum antimicrobial agents.^[^ ^93^ ^]^ Previous studies have reported anti‐bacterial properties of graphene‐based materials via the combined mechanisms of bacterial membrane perturbation caused by sharp edges and oxidative stress induction.^[^ ^94^ ^]^ Additionally, the efficacy of graphene‐based nanocomposites has been investigated with other anti‐microbial agents including metals (mainly silver), metal oxides (e.g., Cu_2_O), photocatalysts (e.g., TiO_2_), quaternary ammonium salts, and polymers (e.g., polypyrrole).^[^ ^95^ ^]^ Since the outbreak of COVID‐19, many companies have rapidly launched graphene‐enhanced PPE by taking the advantage of graphene's antimicrobial, antistatic, and electrically conductive properties to develop face masks with anti‐viral properties which can be re‐sterilised and reused.^[^ ^96^ ^]^ However, there remains lack of evidence behind such claims. Additionally, graphene face covers were removed from Québec's (Canada) schools and day‐care centres after a warning from Canada's national public health agency that inhaling the graphene could lead to asbestos‐like lung damage.^[^ ^97^ ^]^ Nevertheless, a small quantity of graphene and other 2D materials could be compounded with polymers (e.g, PET or PP) (**Figure** **3a**), and then melt‐spun into a polyester yarn or a polypropylene nonwoven matt (Figure 3b) with enhanced mechanical (strength), and improved functional (anti‐viral, fluid repellent, and moisture management) properties. Such fabrics will be used for personal protective clothing to protect healthcare workers and general population from highly infectious diseases including COVID‐19. Furthermore, graphene‐based materials can be functionalised or blended with other functional polymers, and then coated on (Figure 3b), and cross‐linked with textiles to produce durable and washable protective clothing that could potentially mitigate the risk of inhaling graphene.

### 3.2 Filtration

Viruses may be transmitted among human populations via various pathways.^[^ ^98^ ^]^ In the following section, we will discuss the importance of filters in reducing transmission in both air and water systems. In many studies, bacteriophages are used as proxies for viruses under study because they are harmless to humans and a range of them exist with different sizes and surface potentials.

With the exception of hydrogen, the basal plane of graphene has shown to be an impermeable species as small as helium,^[^ ^99^ ^]^ due to the π‐orbitals forming a dense cloud, blocking transport through the aromatic rings. Selective graphene membranes can, however, be formed by forming multi‐layered structures where channels are formed by the interlayer distance between adjacent monolayers. GO has extensively been studied for filtration in both with water^[^ ^100^ ^]^ and gas filtration^[^ ^101^ ^]^ due to its ease of processability and modification of functional groups allowing selectivity to be enhanced. Additionally, the chemical functional groups present in GO change the surface energy and give it a greater stability in water‐based systems than pristine graphene. GO is typically produced by the Hummer's method^[^ ^102^ ^]^ or modified Hummer's method, and production has progressed in recent years towards kiloton scale. GO membranes are produced from an aqueous solution by vacuum filtration, spray coating or bar coating. The resulting lamellar film is composed of GO platelets with a high level of orientation to the plane of the membrane. The effect of orientation on the anti‐bacterial activity has shown that exposing bacteria to edges by vertical orientation increases bacterial decay.^[^ ^103^ ^]^ The lamellar structure defines pathways for water (or other liquids), and gases to pass through the membrane by following a tortuous path around each principal particle. The nature of the chemical functionalisation defines the size and surface charge of the channels which the permeate must pass. The flux and selectivity of the membranes can be modified by chemical treatment of the membranes through chemical or thermal reduction,^[^ ^104^ ^]^ and further functionalisation.^[^ ^101^ ^]^

Coronaviruses can be found in wastewater streams and introduced from hand washing and viral shedding in faces from infected individuals.^[^ ^105^ ^]^ With wastewater streams being a potential transmission pathway, it is necessary to understand the mechanisms for deactivation or removal. Viruses are predominantly removed by size exclusion and modified by the physiochemical properties of the membrane surface. Membranes have been studied for removal of viruses from water streams using conventional membrane materials ultrafiltration membranes^[^ ^106^ ^]^ but are limited by large pore size and limited biofouling resistance. Reverse osmosis membranes are less well studied^[^ ^107^ ^]^ but have lower flux and greater energy requirements. Membrane properties can be modified by embedding of nanoparticles of silver,^[^ ^108^ ^]^ copper and selenium,^[^ ^109^ ^]^ and zinc oxide,^[^ ^110^ ^]^ and these can lead to increases in antiviral efficacy. GO membranes have been suggested for inactivation and removal of bacteria^[^ ^111^ ^]^ from water supplies due to their high water flux and small, defined pore size. While much has been studied about the aerosol transmission of the COVID‐19 virus through respiration, much less is known about its transmission pathway through wastewater streams. Inactivation of viruses in water using GO‐based materials has been studied by several groups^[^ ^112^, ^113^ ^]^ but not in membrane specific use cases.

Inactivation by photocatalysis has been proposed because it is versatile and does not result in addition of further harmful substances to the stream^[^ ^114^, ^115^ ^]^ with TiO_2_ being the most studied material. Other 2D materials with photoactivated processes have been studied for their inactivation efficacy of viruses^[^ ^116^ ^]^ including molybdenum disulphide (MoS_2_) where Mo‐rich edges are responsible for a 10^5^ increase in efficiency over standard MoS_2_ nanosheets under visible light^[^ ^117^ ^]^ and chitosan‐functionalised MoS_2_^[^ ^118^ ^]^ under NIR radiation. These open up the possibility of highly efficient inactivation in wastewater streams using photocatalytic processes.

Monitoring of COVID‐19 in wastewater has been found to correlate with virus circulation in the population^[^ ^119^ ^]^ suggesting that systematic quantification can be used to guide governmental control measures. Various graphene‐based sensing platforms have been studied which could be integrated with wastewater treatment plants for real‐time quantification and planning purposes.^[^ ^120^ ^]^ The potential of graphene to form a simultaneous filtration, inactivation and monitoring membrane may be a direction of future progress worthy of study.

Graphene and GO membranes have also been studied for inactivation of the COVID‐19 virus in aerosols and droplets in air. Masks and face coverings have proven an effective control measure^[^ ^121^ ^]^ in reducing person to person transmission of COVID‐19. Up to 50 particles per second can be produced when a person is talking,^[^ ^122^ ^]^ and these particles include droplets (>5 µm diameter) and aerosols (<5 µm diameter). Droplets, being larger mass carry a higher virus load and present higher rate of transmission but tend to sediment faster than aerosols. Due to their smaller size, aerosols may stay airborne for much longer and build up in poorly ventilated rooms over time, increasing rate of transmission.^[^ ^123^ ^]^ Improving air circulation using air conditioning units has been proposed to reduce airborne viral load and the use of HEPA^[^ ^124^ ^]^ filters^[^ ^125^ ^]^ have been studied as to their effectiveness. Air purification systems which include filters and active measures such as plasma treatment can be an effective method to reduce transmission rate.^[^ ^125^ ^]^

Absorption filters can be used to remove viruses from air circulation systems and include carbon‐based, oxide‐based and ceramic‐based systems. In each of these systems, the electrostatic interactions play roles in the efficacy of the filtration. Activated carbon is a common filtration media and has been shown to be effective at removing a range of contaminants from both air and water systems. Activated carbon is a mesoporous material with a fractal structure of pores which allows high flux and high surface area—both critical factors in determining the suitability of the filter for use. Matsushita et al.^[^ ^126^ ^]^ studied the efficacy of 11 different commercially available activated carbon materials from a variety of feedstocks to study the effect of surface area, elemental contents and functional groups on the efficacy of virus removal. The study found that electrophoretic repulsive forces, pore size distribution, hydrophobicity all contributed to the level of virus removal. With more hydrophobic, lower negative surface charge, high pore volume (20–50 nm) activated carbons being more effective in virus removal.

GO is the most studied 2D material for inactivation of bacteria and viruses. GO interacts with viruses by hydrogen bonding, electrostatic interaction and redox reaction.^[^ ^127^ ^]^ Efficiency can be enhanced by creating hybrid structures such as GO‐silver composites enhance natural antiviral defences. Palmieri and Papi^[^ ^128^ ^]^ provided a review in the early phase of the COVID‐19 pandemic and discuss various inactivation mechanisms of GMs with viruses. High surface area carbon materials like graphene provide a good scaffold for novel virus inhibitors, creating a versatile platform filter.

## 4 Outlook

The COVID‐19 pandemic highlights the need for rapidly available tools and technologies for detection, protection, and continuous monitoring of viruses. Owing to their unique physicochemical properties, graphene and graphene‐like other 2DM could play a vital role in combating COVID‐19 and other future viruses. The excellent electrical, mechanical, thermal, and optical properties make them ideal candidate for manufacturing biosensors, PPE, wearable electronics, and filtration techniques to detect and protect from viruses. Biosensors could potentially be used for rapid, low‐cost, and mass level testing, enabling self‐isolation for infectious people. 2DM could be used for the development of such biosensors for virus detection with extremely small dimensions, due to their smaller size and excellent physiochemical properties of 2DM. Additionally, 2DM‐based biosensors offer great performance in terms of selectivity and sensitivity. The layered 2D structures combined with ion‐conductive polymers, polyelectrolytes, can convert the chemical energy into electrical energy and electrical energy into chemical energy. The change of ionic concentration in the interior of the layered composite material can enhance the efficiency of bio‐detection. A more complex structure with several different layers which are differently charged can be self‐assembled into bipolar ionic transistors. Such structures can show intrinsic amplification of the signal, increasing sensitivity of biosensor.^[^ ^69^ ^]^ Furthermore, recent progress in 2DM‐based printed electronics will offer low‐cost and large‐scale fabrication of printed biosensors.^[^ ^129^ ^]^

Wearable electronics has demonstrated great potential for personalised healthcare applications by monitoring physiological conditions. Graphene and other 2DM offer truly multifunctional wearable electronics platforms that could potentially monitor patients’ vital signs continuously in a non‐invasive way. Graphene‐based wearable electronic devices can be used in the early detection of asymptomatic and pre‐symptomatic cases of COVID‐19 and play a vital role in combating the next pandemic. However, successful realisation of robust and reliable medical‐grade wearables will require collaborative joint‐efforts from materials scientists, electrical engineers, data scientists, commercial partners, and healthcare providers.

There has been unprecedented demand and huge shortage of PPE to protect the health of HCWs and prevent the spread of COVID‐19 viruses. The critical shortage of PPE supply has been one of major causes for healthcare workers death during COVID‐19 pandemic. There must be efforts from researchers and manufacturers of PPE to improve the performance and safety with existing PPE. Graphene, its derivatives and other 2DM could potentially improve the anti‐viral performance of PPE, and make them stronger, lighter, and more comfortable to wear for HCWs. Additionally, the huge surge in the use of environmentally unfriendly plastic‐based protective clothing during COVID‐19 pandemic has been a great concern to the environmentalists. Therefore, many efforts are needed to develop either biodegradable or recycled materials‐based PPE. The coating or compounding of graphene and other 2DMs with such natural or recycled materials can improve mechanical and functional properties.^[^ ^13^, ^130^ ^]^

Graphene and 2DMs have shown promise in filtration and inactivation of viruses through forming nanocomposite filters and coatings with conventional materials, where their physiochemical and photocatalytic behaviour allows viruses to be inactivated. Such filtration devices could be used in air‐purification and air‐conditioning machines to efficiently filter clean the COVID‐19 and other respire germs present in air. Additionally, 2DM‐based membranes could be used to separate COVID‐19 viruses from water by tuning their microstructural properties and interlayer spacing. Furthermore, 2DMs‐based photocatalyst can be used for inactivation and degradation of COVID‐19 in water directly. Therefore, further work is needed to develop next generation smart and sustainable graphene‐based membrane that could not only filter the viruses but also destroy and sterilise the water.

Graphene‐based composite materials have shown promise for antiviral treatment by inhibiting viruses from entering the host cells. In a previous study silver nanoparticle‐modified GO (GO‐AgNPs) nanocomposites was used as an antiviral agent to treat highly infectious porcine reproductive and respiratory syndrome virus (PRRSV). Such antimicrobial composites provide a broad antiviral activity including 59.2% inhibition efficiency against PRRSV.^[^ ^131^ ^]^ In another study, sulfonated magnetic nanoparticles functionalized with reduced GO was used to capture and photothermally destroy herpes simplex virus type 1 (HSV‐1) using near‐infrared (NIR) light with ≈99.99% photothermal antiviral activity,^[^ ^132^ ^]^ which could be used for NIR treatment of lungs. However, based on these initial findings, future work will be needed to develop graphene‐based antiviral treatments for COVID‐19. Like any other new materials, indeed there has been safety and toxicity concerns with graphene and other 2D materials‐based technologies and products, which need to be carefully addressed and mitigated for specific commercial applications.^[^ ^133^, ^134^ ^]^

## Conflict of Interest

The authors declare no conflict of interest.
